# The Choice of Either Quetiapine or Aripiprazole as Augmentation Treatment in a European Naturalistic Sample of Patients With Major Depressive Disorder

**DOI:** 10.1093/ijnp/pyab066

**Published:** 2021-10-12

**Authors:** Lucie Bartova, Gernot Fugger, Markus Dold, Alexander Kautzky, Marleen Margret Mignon Swoboda, Dan Rujescu, Joseph Zohar, Daniel Souery, Julien Mendlewicz, Stuart Montgomery, Chiara Fabbri, Alessandro Serretti, Siegfried Kasper

**Affiliations:** 1 Department of Psychiatry and Psychotherapy, Medical University of Vienna, Vienna,Austria; 2 Department of Biomedical and NeuroMotor Sciences, University of Bologna, Bologna,Italy; 3 Psychiatric Division, Chaim Sheba Medical Center , Tel Hashomer, Israel; 4 School of Medicine, Free University of Brussels, Brussels,Belgium; 5 Psy Pluriel - European Centre of Psychological Medicine, Brussels,Belgium; 6 Imperial College School of Medicine, University of London, London,United Kingdom; 7 Social, Genetic and Developmental Psychiatry Centre, Institute of Psychiatry, Psychology and Neuroscience, King’s College London, London,United Kingdom; 8 Center for Brain Research, Medical University of Vienna, Vienna,Austria

**Keywords:** Antidepressant treatment, aripiprazole, augmentation, major depressive disorder, quetiapine, second-generation antipsychotics

## Abstract

**Background:**

Augmentation with second-generation antipsychotics (SGAs) represents an evidence-based psychopharmacotherapeutic strategy recommended in case of insufficient response to the first-line antidepressant (AD) treatment in major depressive disorder (MDD). Comparative evidence regarding efficacy and prescription preferences of the individual SGAs is scarce.

**Methods:**

In the scope of this European, multi-site, naturalistic cross-sectional investigation with retrospective assessment of treatment outcome, we compared sociodemographic and clinical characteristics of 187 MDD patients receiving either quetiapine (n = 150) or aripiprazole (n = 37) as augmentation of their first-line AD psychopharmacotherapy.

**Results:**

Comorbid posttraumatic stress disorder and diabetes were significantly associated with aripiprazole augmentation in our primary and post-hoc binary logistic regression analyses. Furthermore, we identified an association between aripiprazole co-administration and the presence of additional psychotic features, higher rates of AD combination treatment, and a longer duration of psychiatric hospitalizations during the lifetime, which, however, lost significance after correcting for multiple comparisons. Regarding treatment outcome, we found a trend of higher response rates and greater reductions in severity of depressive symptoms in MDD patients dispensed quetiapine.

**Conclusions:**

Factors associated with a more chronic and severe profile of MDD seem to encourage clinicians to choose aripiprazole over quetiapine, that was, however, administered in the majority of our MDD patients, which might reflect the current approval situation allowing to prescribe exclusively quetiapine as on-label augmentation in MDD in Europe. Given the retrospective assessment of treatment response, the markedly smaller proportion of patients receiving aripiprazole augmentation generally showing an unfavorable disease profile, and the partially heterogeneous statistical robustness of our findings, further studies are required to elaborate on our observation and to generate unambiguous recommendations regarding the choice of first-line SGA augmentation in MDD.

**Significance Statement**

In line with recommendations of most clinical practice guidelines (CPG), second-generation antipsychotics (SGAs) are frequently administered as additional psychopharmacotherapeutic approach in case of insufficient treatment outcome with the first-line antidepressant therapy in major depressive disorder (MDD). However, there is a paucity of evidence revealing which individual SGA suits best for a given patient. Our naturalistic, cross-sectional multicenter data of 187 MDD patients revealed that the majority (n = 150) was prescribed quetiapine, which was associated with a more beneficial disease profile. Concurrently, a more chronic and severe illness profile, especially the presence of psychiatric and somatic comorbidities and trend-wise additional psychotic features, higher depression severity, the requirement of polypharmacy, worse treatment outcome during the current MDE, as well as a longer lifetime-period spent in psychiatric inpatient-care, may represent factors that encourage clinicians to prefer aripiprazole. The aforementioned contrasts may serve as basis for future longitudinal research aiming at individually tailored MDD treatments.

## Introduction

Approximately two-thirds of the patients suffering from major depressive disorder (MDD) fail to adequately respond and/or achieve remission with their first-line antidepressant (AD) psychopharmacotherapy and require additional treatment strategies (Kolovos et al., 2017). The administration of second-generation antipsychotics (SGAs) represents one of the most frequently applied and rigorously investigated psychopharmacotherapeutic approaches (Konstantinidis et al., 2012; Wang et al., 2016; Gerhard et al., 2018). Due to its efficacy and tolerability, most international treatment guidelines recommend SGA augmentation as the first-line psychopharmacotherapeutic option in case of insufficient response in MDD (Bauer et al., 2017; Dold and Kasper, 2017). Hereby, the efficacy of individual SGAs, when administered as augmenting agents to the first-line AD therapy in MDD, has been evidenced by numerous randomized-controlled trials (RCTs), open label trials, and meta-analyses (Spielmans et al., 2013; Han et al., 2014; Lin et al., 2014; Wang et al., 2015; Zhou et al., 2015; Mohamed et al., 2017). In Europe, quetiapine extended release (XR) represents the only SGA approved for the abovementioned indication by regulatory authorities. In the United States, this is true for quetiapine XR, aripiprazole, the combination of fluoxetine and olanzapine, and brexpiprazole (Wang et al., 2016). A large network meta-analysis indicated that standard daily dosages of quetiapine, aripiprazole, olanzapine, and risperidone are equally effective options to augment AD psychopharmacotherapy in case of insufficient response in MDD (Zhou et al., 2015). However, the 2 SGAs—aripiprazole and quetiapine—are supported by especially strong international data (Connolly and Thase, 2011).

The XR formulation of quetiapine has not only been shown to be potent in augmenting first-line AD therapy in MDD (Weisler et al., 2013) but has also proven efficacy when administered as AD monotherapy (Weisler et al., 2012; Montgomery et al., 2014). Such robust AD effects were suggested to be mediated via serotonin (5-HT) 1A and 2A, dopamine, and glutamate receptors as well as norepinephrine transporters (Pae et al., 2010). Aripiprazole, representing the first SGA that obtained approval for augmentation in MDD by the US Food and Drug Administration (Wang et al., 2016; Mohamed et al., 2017), stands out from most other SGAs as it exhibits a partial dopamine-2 (D2) receptor agonism, allowing the so-called “dopamine stabilization.” Together with its potency of partial agonism at the 5-HT1A receptor, these properties make aripiprazole particularly interesting for MDD treatment (Frankel and Schwartz, 2017). Furthermore, in a recent meta-analysis focusing on augmentation strategies in treatment-resistant depression (TRD) defined by insufficient response to at least 2 ADs, aripiprazole reached the highest therapeutic effect size among all investigated SGAs in this indication (Strawbridge et al., 2019).

In light of the dearth of studies comparing augmentation of the first-line AD psychopharmacotherapy with either quetiapine or aripiprazole in MDD, we sought to enrich the existing evidence by specifically addressing 2 clinically relevant research questions in the course of the present study. Firstly, we aimed to elucidate factors potentially mediating the clinicians’ decision of prescribing either quetiapine or aripiprazole augmentation by systematically comparing the socio-demographic and clinical characteristics of MDD patients dispensed either compound. Our second goal was to elucidate differences in treatment outcome patterns related to the prescription of quetiapine or aripiprazole, respectively.

## METHODS

### Study Design

The present work represents a secondary analysis of an international, multicenter, observational, cross-sectional, and non-interventional study with a retrospective evaluation of treatment outcome that was performed by the European Group for the Study of Resistant Depression (GSRD) (Bartova et al., 2019). These post-hoc analyses refer to the project “Clinical and biological correlates of resistant depression and related phenotypes” conducted between 2011 and 2016 by 10 research centers located in Austria, Belgium, France (2 sites), Germany, Greece, Israel, Italy (2 sites), and Switzerland (Dold et al., 2016, 2018; Bartova et al., 2019). Local ethics committees of the abovementioned research centers approved the study design and all study procedures that were introduced previously (Dold et al., 2018; Bartova et al., 2019) and are, hence, provided in a compendious way here.

### Study Collective

The recruitment of adult, male and female in- and outpatients was performed in both university as well as non-academic clinical routine centers in 8 European countries that are mentioned above. Patients who were eligible to study participation signed written informed consent after a thorough explanation of the study procedures. A present single or recurrent major depressive episode (MDE) occurring in the course of MDD that was diagnosed based on the DSM-IV-TR criteria (Wittchen et al., 1997) and represented the primary psychiatric diagnosis was mandatory for study enrollment. An ongoing and adequate psychopharmacotherapy comprising a first-line AD agent that was administered in sufficient daily doses at least for 4 weeks during the current MDE represented further inclusion criterion (Dold et al., 2016; Bartova et al., 2019). Moreover, augmentation treatment with either quetiapine (first-line AD agent + quetiapine) or aripiprazole (first-line AD agent + aripiprazole) was required. Concerning the SGA quetiapine, a daily dose of ≥100 mg/d was determined as minimum to ensure that quetiapine was administered as augmentation treatment and to avoid enrollment of patients treated with low-dose quetiapine for symptoms such as sleep disturbance, agitation, and/or anxiety (Dold et al., 2018). Regarding aripiprazole, a daily administration of minimally 2.5 mg was mandatory for this compound to be considered as augmentation treatment. Any primary psychiatric diagnosis other than MDD as well as comorbid substance use disorders (with exception of caffeine and nicotine) present in the previous 6 months and/or severe personality disorders represented exclusion criteria. Other psychiatric as well as somatic comorbidities and the presence of specific disease manifestations occurring during the current MDE such as psychotic and/or melancholic features and/or suicidality were allowed in the course of the naturalistic character of the present study (Dold et al., 2018; Bartova et al., 2019).

### Clinical Evaluation

Socio-demographic, clinical, and treatment patterns of MDD patients were evaluated exclusively by experienced and specifically trained psychiatrists. In the course of this comprehensive clinical assessment, medical records of the patients were considered and the Mini International Neuropsychiatric Interview (Sheehan et al., 1998) was performed to establish the primary psychiatric diagnosis, the presence of additional specific features occurring during the current MDE, as well as potential psychiatric and/or somatic comorbidities. Furthermore, treatment strategies employed during the current MDE were rigorously evaluated. The severity of depressive symptoms at study initiation, reflecting a time-point after at least 4 weeks of adequate AD psychopharmacotherapy, was measured using the 21-item Hamilton Rating Scale for Depression (Hamilton, 1960) and the Montgomery and Åsberg Depression Rating Scale (MADRS; current MADRS, cMADRS) (Montgomery and Asberg, 1979). The severity of depressive symptoms at the onset of the current MDE, reflecting a time-point before the first-line AD treatment was initiated, which was minimally 4 weeks before study enrollment, was assessed employing the retrospective MADRS (rMADRS) calculated according to the MDD patients’ assertions together with clinical data derived from the medical records of the patients. All ratings were performed exclusively by experienced psychiatrists undergoing specific rater trainings to assure a high standard of inter-rater reliability.

Based on the GSRD staging model for treatment outcome, the MADRS total score change (retrospective MADRS – cMADRS) was gathered after at least 1 adequate AD trial administered at sufficient daily dosages for at least 4 weeks (Bartova et al., 2019). Briefly, treatment response was characterized by a cMADRS total score of <22 and a ≥50% reduction of the MADRS total score after an adequate AD trial lasting minimally 4 weeks. Non-response to AD treatment was defined as a total score of ≥22 at the cMADRS and a <50% MADRS total score reduction after 1 AD trial of adequate daily dosing and duration. Treatment resistance was categorized as a non-response to 2 or more consecutive AD trials of adequate daily dosing and duration (Bartova et al., 2019).

### Statistical Procedure

All eligible MDD patients derived from a subject pool of the GSRD (Dold et al., 2018; Bartova et al., 2019) were subdivided into 2 groups based on whether they underwent augmentation treatment with either quetiapine or aripiprazole that was administered additionally to their ongoing first-line AD psychopharmacotherapy. MDD patients who simultaneously received both augmentations strategies were excluded from these post-hoc analyses performed with version 27 of IBM SPSS Statistics.

The related socio-demographic, clinical, and psychopharmacotherapeutic patterns were dichotomously compared between the 2 patient groups and are depicted with descriptive statistics (means, SD, and/or percentages) in Table 1. Between-group differences analyzed using chi-squared tests for categorical variables and ANCOVAs for continuous variables with the respective augmentation treatment as fixed effect and recruitment center as covariate are also displayed in Table 1. Hereby, we employed the Bonferroni-Holm correction for multiple comparisons. In case of statistical significance that was set at *P* ≤ .05, binary logistic regression analyses with the relevant independent variables were conducted to analyze their relation to the employed augmentation treatment with either quetiapine or aripiprazole representing the dichotomous dependent variable, whereby the recruitment center served as covariate (Table 2).

**Table 1.** Socio-Demographic, Clinical, and Treatment Correlates of Augmentation Treatment with Either Quetiapine or Aripiprazole in 187 MDD Patients

MDD patients’ characteristics	Total sample (n = 187)	Augmentation with quetiapine (n = 150)	Augmentation with aripiprazole (n = 37)	x^2^/F	*P* (x^2^/ANCOVA)
Sex, n (%)					
Female	116 (62.0)	92 (61.3)	24 (64.9)	0.157	.692
Male	71 (38.0)	58 (38.7)	13 (35.1)		
Age, mean (SD), y (n = 186)	51.9 (13.0)	51.9 (12.4)	52.1 (15.5)	0.025	.876
Bodyweight, mean (SD), kg (n = 186)	79.4 (18.2)	78.7 (18.6)	81.9 (16.6)	0.798	.373
Ethnicity, n (%)					
Caucasian origin	185 (98.9)	148 (98.7)	37 (100.0)	0.499	.480
Educational level, n (%) (n = 186)					
University education/non-university high education/high level general education	77 (41.4)	63 (42.3)	14 (37.8)	0.241	.623
General secondary/technical education/elementary school/none	109 (58.6)	86 (57.7)	23 (62.2)		
Occupational status, n (%) (n = 185)					
Employed	50 (27.0)	41 (27.5)	9 (25.0)	0.093	.760
Unemployed	135 (73.0)	108 (72.5)	27 (75.0)		
Relationship status, n (%)					
With ongoing relationship	98 (52.4)	79 (52.7)	19 (51.4)	0.021	.886
Without ongoing relationship	89 (47.6)	71 (47.3)	18 (48.6)		
Disease course, n (%)					
Single MDD episode	10 (5.3)	10 (6.7)	0 (0.0)	2.606	.106
Recurrent MDD	177 (94.7)	140 (93.3)	37 (100.0)		
Additional features during the current MDD episode, n (%)					
Psychotic features	34 (18.2)	23 (15.3)	11 (29.7)	4.135	.042
Melancholic features	152 (81.3)	120 (80.0)	32 (86.5)	0.821	.365
Atypical features	10 (5.3)	10 (6.7)	0 (0.0)	2.606	.106
Catatonic features	0 (0.0)	0 (0.0)	0 (0.0)	—	—
Suicidality^*a*^					
Current suicidal risk (dichotomous)	110 (58.8)	87 (58.0)	23 (62.2)	0.212	.645
High/moderate	59 (53.6)	46 (52.9)	13 (56.5)	0.097	.755
Low	51 (46.4)	41 (47.1)	10 (43.5)		
Treatment setting, n (%)					
Inpatient	124 (66.3)	98 (65.3)	26 (70.3)	0.324	.569
Outpatient	63 (33.7)	52 (34.7)	11 (29.7)		
Chronicity					
Duration of current MDD episode, mean (SD), d (n = 154)	179.6 (160.7)	177.5 (158.9)	187.8 (170.1)	0.130	.719
No. of MDD episodes during lifetime, mean (SD) (n = 153)	3.4 (2.4)	3.2 (2.1)	4.1 (3.4)	2.930	.089
Age of disease onset, mean (SD), y (n = 179)	35.8 (14.4)	36.2 (14.0)	34.2 (16.0)	0.364	.547
Duration of psychiatric hospitalizations during lifetime, mean (SD), wk (n = 174)	13.6 (30.1)	10.8 (20.8)	24.4 (51.2)	6.341	.013
Psychiatric comorbidities, n (%)					
Any anxiety disorder	41 (21.9)	34 (22.7)	7 (18.9)	0.244	.622
Generalized anxiety disorder	20 (10.7)	17 (11.3)	3 (8.1)	0.323	.570
Panic disorder	20 (10.7)	17 (11.3)	3 (8.1)	0.323	.570
Agoraphobia	22 (11.8)	16 (10.7)	6 (16.2)	0.881	.348
Social phobia	7 (3.7)	7 (4.7)	0 (0.0)	1.794	.180
Obsessive-compulsive disorder (n = 184)	4 (2.2)	4 (2.7)	0 (0.0)	0.995	.319
Posttraumatic stress disorder	6 (3.2)	2 (1.3)	4 (10.8)	8.584	**.003**
Somatic comorbidities, n (%)					
Any somatic comorbidity	99 (52.9)	80 (53.3)	19 (51.4)	0.047	.829
Hypertension	51 (27.3)	39 (26.0)	12 (32.4)	0.619	.431
Thyroid dysfunction	43 (23.0)	31 (20.7)	12 (32.4)	2.320	.128
Migraine	18 (9.6)	14 (9.3)	4 (10.8)	0.074	.785
Diabetes	15 (8.0)	8 (5.3)	7 (18.9)	7.425	**.006**
Heart disease	17 (9.1)	14 (9.3)	3 (8.1)	0.054	.816
Arthritis	7 (3.7)	7 (4.7)	0 (0.0)	1.794	.180
Asthma	8 (4.3)	6 (4.0)	2 (5.4)	0.143	.705
Pain	1 (0.5)	1 (0.7)	0 (0.0)	0.248	.618
Severity of depressive symptoms, mean (SD)					
HAM-D total 21-item at study entry	21.1 (8.8)	20.7 (9.1)	23.0 (7.6)	1.813	.180
MADRS total at study entry (cMADRS)	27.2 (11.2)	26.4 (11.5)	30.5 (8.9)	3.817	.052
MADRS total at onset of the current MDE (rMADRS)	36.9 (8.1)	36.5 (8.4)	38.6 (7.0)	1.889	.171
Treatment outcome, n (%)^*b*^					
Response	39 (20.9)	35 (23.3)	4 (10.8)	5.983	.050
Non-response	64 (34.2)	54 (36.0)	10 (27.0)		
Resistance	84 (44.9)	61 (40.7)	23 (62.2)		
MADRS total score change (rMADRS - cMADRS), mean (SD)	−9.7 (11.0)	−10.1 (11.9)	−8.1 (5.8)	0.854	.357
Ongoing psychotherapy, n (%) (n = 162)					
Any psychotherapy	58 (35.8)	44 (34.4)	14 (41.2)	0.541	.462
Cognitive behavioral therapy	40 (24.7)	32 (25.0)	8 (23.5)	3.393	.494
Psychoanalytic psychotherapy	6 (3.7)	5 (3.9)	1 (2.9)		
Systemic psychotherapy	5 (3.1)	3 (2.3)	2 (5.9)		
Other psychotherapy	7 (4.3)	4 (3.1)	3 (8.8)		
Ongoing psychopharmacotherapy					
Number of concurrently administered psychopharmacotherapeutics, mean (SD)	3.5 (1.0)	3.4 (1.0)	3.7 (1.0)	2.286	.132
Administered first-line antidepressant in the current MDD episode, n (%)					
Selective serotonin reuptake inhibitors	80 (42.8)	61 (40.7)	19 (51.4)	10.7	.153
Serotonin-norepinephrine reuptake inhibitors	64 (34.2)	53 (35.3)	11 (29.7)		
Noradrenergic and specific serotonergic antidepressants	16 (8.6)	15 (10.0)	1 (2.7)		
Tricyclic antidepressants	15 (8.0)	13 (8.7)	2 (5.4)		
Agomelatine	1 (0.5)	0 (0.0)	1 (2.7)		
Noradrenaline-dopamine reuptake inhibitors	7 (3.7)	4 (2.7)	3 (8.1)		
Serotonin antagonist and reuptake inhibitors	3 (1.6)	3 (2.0)	0 (0.0)		
Monoamine oxidase inhibitors	1 (0.5)	1 (0.7)	0 (0.0)		
Noradrenaline reuptake inhibitors	0 (0.0)	0 (0.0)	0 (0.0)		
Vortioxetine	0 (0.0)	0 (0.0)	0 (0.0)		
Tianeptine	0 (0.0)	0 (0.0)	0 (0.0)		
Daily doses given in fluoxetine equivalents,^*c*^ mean (SD), mg/d (n = 155)	51.5 (21.8)	51.4 (22.8)	51.7 (17.0)	0.000	.985
Further augmentation/combination strategies administered together with the ongoing antidepressanttreatment, n (%)					
Combination with at least 1 additional antidepressant	92 (49.2)	68 (45.3)	24 (64.9)	4.530	.033
Augmentation with at least 1 mood stabilizer	25 (13.4)	17 (11.3)	8 (21.6)	2.712	.100
Augmentation with pregabalin	27 (14.4)	21 (14.0)	6 (16.2)	0.118	.731
Augmentation with at least 1 low-potency antipsychotic^*d*^	9 (4.8)	7 (4.7)	2 (5.4)	0.035	.851
Augmentation with benzodiazepines including zolpidem and zopiclone	96 (51.3)	82 (54.7)	14 (37.8)	3.365	.067

Abbreviations: HAM-D, Hamilton Depression Rating Scale; MADRS, Montgomery Åsberg Depression Rating Scale (cMADRS, current MADRS; rMADRS, retrospective MADRS); MDD, major depressive disorder; MDE, major depressive episode.

The *P* values displayed in bold were signi/cant after Bonferroni-Holm correction.

^*a*^The presence of the current suicidal risk was measured based on the HAM-D item 3 (suicidality) ratings, whereby the item-score 1 characterized low and the item-scores 2 to 4 moderate to high degree of the current suicidal risk (Dold et al., 2018b).

^*b*^Non-response was defined by a previous single failed trial and treatment resistance by 2 or more failed trials.

^*c*^Fluoxetine dose equivalents were calculated according to Hayasaka et al. (2015).

^*d*^Low-potency antipsychotics comprise the so-called low-potency first-generation antipsychotics and the second-generation antipsychotic quetiapine <100 mg/d (Dold et al., 2016).

**Table 2.** Binary Logistic Regression Analyses Displaying Associations of the Augmentation Treatments With Significant Variables in Our Primary Analyses

MDD patients’ characteristics	Adjusted OR (95% CI)	*P*
Comorbid posttraumatic stress disorder	0.112 (0.020–0.639)	.014
Comorbid diabetes	0.240 (0.081–0.714)	.010

Abbreviations: CI = confidence interval; MDD = major depressive disorder; OR = odds ratio.

Table 2 displays results of our post-hoc binary logistic regression analyses on the association between the administered augmentation treatment with either quetiapine or aripiprazole and variables identified as significant in our primary analyses in 187 MDD patients. The present analyses were adjusted for the variable research center. Adjusted ORs with 95% CIs are presented for these 2 dichotomous independent variables.

## RESULTS

**Figure 1.**
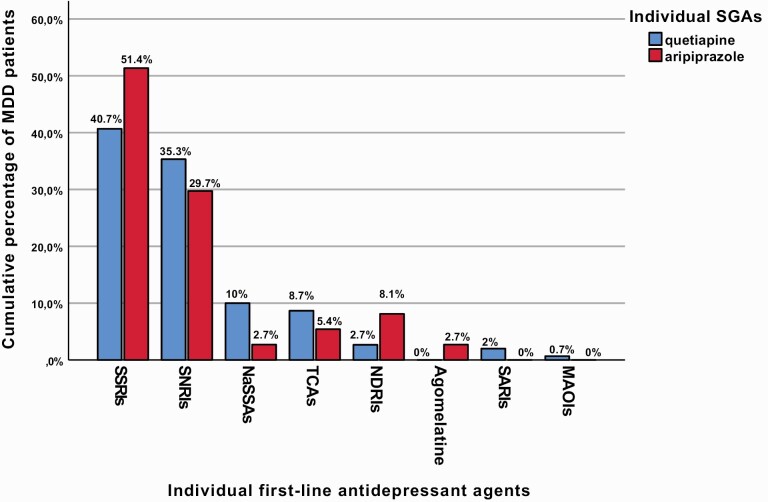
First-line antidepressant (AD) treatment administered in major depressive disorder (MDD) patients receiving augmentation treatment with either quetiapine or aripiprazole. Displayed cumulative percentages refer to the first-line AD treatment administered in 187 MDD patients receiving augmentation with either quetiapine (n = 150; blue colored) or aripiprazole (n = 37; red colored), whereby no significant between-group differences were detected (*P* = .153). Abbreviations: MAOIs, monoamine oxidase inhibitors; NARIs, noradrenaline reuptake inhibitors; NaSSAs, noradrenergic and specific serotonergic ADs; NDRIs, noradrenergic-dopamine reuptake inhibitors; SARIs, serotonin antagonist and reuptake inhibitors; SGAs, second-generation antipsychotics; SNRIs, serotonin- norepinephrine reuptake inhibitors; SSRIs, selective serotonin reuptake inhibitors; TCAs, tricyclic ADs.

In total, the analyzed sample included 187 MDD patients, whereby 150 (80.2%) received augmentation with quetiapine and 37 (19.8%) with aripiprazole, both of which were administered together with the first-line AD psychopharmacotherapy (Figure 1). The socio-demographic, clinical, and therapeutic characteristics of the whole sample and both patient groups itemized according to their augmentation treatment with either quetiapine or aripiprazole as well as the identified between-group contrasts are displayed in Table 1 in detail. Table 2 depicts the results of our post-hoc binary logistic regression analyses on the association between the administered augmentation treatment and variables for which significant between-group differences were detected in our initial analyses. Significant and clinically meaningful results of the abovementioned analyses are summarized below, whereby exclusively statistical parameters of our primary analyses (Table 1) are provided.

**Figure 2.**
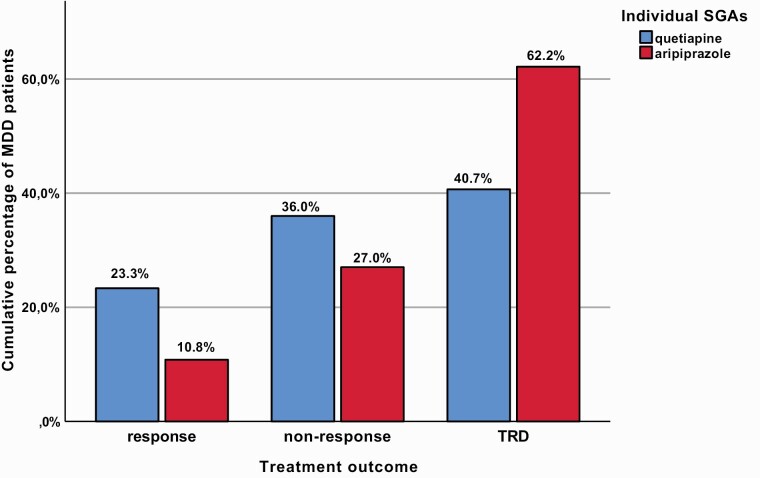
Treatment outcome pattern trends in major depressive disorder (MDD) patients receiving augmentation treatment with either quetiapine or aripiprazole. Displayed cumulative percentages refer to the proportion of MDD patients (n = 187) receiving augmentation treatment with either quetiapine (n = 150; blue colored) or aripiprazole (n = 37; red colored) that are itemized according to their treatment outcome patterns subdivided into 3 groups (response, non-response, treatment-resistant depression [TRD]). While non-response was defined by a previous single failed adequate antidepressant trial, at least 2 failed adequate antidepressant trials were mandatory for TRD (Bartova et al., 2019). The presented result of our between-group analyses on treatment outcome reached uncorrected borderline significance (*P* = .050). Abbreviations: SGAs, second-generation antipsychotics.

Compared with MDD patients treated with quetiapine augmentation, comorbid posttraumatic stress disorder (PTSD; 1.3% vs 10.8%, *P* = .003) and diabetes mellitus (DM; 5.3% vs 18.9%, *P* = .006) occurred more frequently in individuals with aripiprazole augmentation treatment who were hospitalized in psychiatric inpatient units longer during their lifetime (10.8 ± 20.8 vs 24.4 ± 51.2 weeks, *P*_uncorrected_ = .013). During the current MDE, additional psychotic features appeared more frequently in MDD patients augmented with aripiprazole (15.3% vs 29.7%, *P*_uncorrected_ = .042) who were also administered an overall higher number of AD agents in the course of an AD combination treatment (45.3% vs 64.9%, *P*_uncorrected_ = .033). In terms of treatment outcome, a trend-wise higher proportion of responders to AD treatment was identified in MDD patients receiving quetiapine augmentation (23.3% vs 10.8%), while resistance to AD treatment occurred more commonly in MDD patients augmented with aripiprazole (40.7% vs 62.2%, *P*_uncorrected_ = .050; Figure 2). A trend towards greater depression severity at study entry evidenced by the total cMADRS score (26.4 ± 11.5 vs 30.5 ± 8.9, *P*_uncorrected_ = .052) was observed in the group of patients augmented with aripiprazole.

## Discussion

To the best of our knowledge, the current naturalistic, multicenter, cross-sectional investigation is the first that directly compared socio-demographic, clinical, and treatment patterns of MDD patients receiving either quetiapine or aripiprazole, 2 SGAs with the most evidence for augmenting the ongoing AD first-line therapy in case of insufficient response in MDD (Connolly and Thase, 2011; Cantu et al., 2021). In the total sample consisting of 187 European patients with MDD as their primary diagnosis, augmentation treatment with quetiapine was employed in 150 patients, whereas aripiprazole was administered in a relatively small proportion comprising 37 patients. Our most robust findings indicate that aripiprazole was the augmenting agent of choice in MDD patients with comorbid PTSD and DM. Furthermore, a trend towards greater severity of depressive symptoms at study entry, presence of additional psychotic features, employment of AD combination treatment, and higher rates of treatment resistance during the current MDE as well as a longer duration of psychiatric hospitalizations during the lifetime was identified in MDD patients augmented with aripiprazole in relation to those patients receiving quetiapine augmentation of their ongoing AD first-line therapy.

Because every second MDD patient was shown to concurrently receive more than 2 psychopharmacotherapeutics (Dold et al., 2016; Rhee and Rosenheck, 2019), the employment of add-on strategies in general represents a common clinical practice in MDD patients non-responding to their first-line AD treatment. Hereby, the broad range of available add-on psychopharmacotherapies, as well as the diverging evidence-based foundation and potential to promote adverse effects between the individual substances (Mojtabai and Olfson, 2010; Govaerts et al., 2021), have to be considered in the course of the clinical decision process of which augmenting agent to privilege in an individual patient. The fact that quetiapine was applied in the majority of our MDD patients might reflect the adherence of European psychiatrists to the current approval situation in Europe allowing to prescribe exclusively quetiapine XR as on-label augmentation in MDD. The observed administration rates might differ from comparable patient populations recruited in other countries, where a broader spectrum of individual SGAs is licensed (Wang SM et al., 2016; Mohamed et al., 2017). Our results on the employment of further add-on substances revealing solely a trend-wise higher co-administration of aripiprazole and AD combination treatments might point at a rather cautious attitude towards further polypsychopharmacotherapeutic approaches that might have been intensified due to the fact that AEs of antipsychotics appeared to emerge at an increased level when co-administered with further psychopharmacotherapeutics (Iversen et al., 2018).

The fact that aripiprazole augmentation was more commonly applied in MDD patients with comorbid DM in our sample might be due to its favorable metabolic side-effect profile and a lower risk of weight gain (Bak et al., 2014) that represents one of the most common sufferings in the course of comorbid DM with potential negative implications on both DM and MDD (Fugger et al., 2019). It is noteworthy in this regard that quetiapine was associated with a moderate risk of weight gain (Bak et al., 2014), which was, however, still double that of the placebo arms. Somnolence, dizziness, and/or dry mouth were adverse effects more frequently encountered (Pae et al., 2010). Given that many investigations found an association between DM and the administration of antipsychotic agents in general, which was greatly dependent on the substance and the exposure time (Kessing et al., 2010), evidence linking quetiapine or aripiprazole to DM risk or DM complications remains largely inconsistent. While no significant risk difference between quetiapine or aripiprazole prescription was reported in several previous studies (Kessing et al., 2010; Xing et al., 2018), other authors demonstrated that aripiprazole was the only antipsychotic agent that was not associated with a higher incidence of DM (Vancampfort et al., 2016). The latter observation might underline the present results revealing a higher proportion of aripiprazole administrations in patients suffering from MDD and comorbid DM, especially when the overall lower prescription rates of aripiprazole as compared with quetiapine in our patient population are considered.

Despite the identified association between aripiprazole augmentation and the presence of comorbid PTSD, it is noteworthy that firstly, the number of affected patients in our study is very low, which has to be taken into account when interpreting this finding. Secondly, available evidence recommending either agent for the treatment of co-occurring PTSD and MDD is missing. Although a systematic review evaluating aripiprazole monotherapy as treatment option for PTSD in predominantly veteran patient populations found arguments in favor of this SGA (Britnell et al., 2017), the generalizability of the results in primary MDD patients with comorbid PTSD is questionable. On the other hand, a recent large network meta-analysis on the first-line psychopharmacotherapy of PTSD recommended quetiapine monotherapy upon selective 5-HT reuptake inhibitors, venlafaxine, topiramate, and risperidone (de Moraes Costa et al., 2020). Supporting evidence for the efficacy of quetiapine augmentation stems from a single RCT with 80 patients suffering from military-associated PTSD (Villarreal et al., 2016). Given the heterogeneity of the currently available findings and the comparably small number of patients with comorbid PTSD in our study, replications in different and larger patient populations are necessary.

Beyond its associations with the presence of comorbid PTSD and DM, aripiprazole augmentation was trend-wise related to the presence of additional psychotic symptoms, a higher MADRS total score at study entry, the employment of AD combination treatment, and higher rates of treatment resistance during the current MDE as well as a longer duration of inpatient hospitalizations (24.4 vs 10.8 weeks in cases of augmentation with quetiapine) and a higher number of MDEs during the lifetime (4.1 vs 3.2 in cases of augmentation with quetiapine), even though the latter finding did not reach statistical significance (*P*_uncorrected_ = .089). Because the aforementioned clinical characteristics were repeatedly related to disease severity and chronicity and, hence, TRD (Bartova et al., 2019) and the so-called difficult-to-treat depression (McAllister-Williams et al., 2020), aripiprazole seemed to be preferably administered in MDD patients with rather unfavorable disease and treatment outcome patterns. With respect to the observed effects of aripiprazole and quetiapine on treatment outcome per se, our analyses, however, gently point towards a benefit of quetiapine augmentation. In fact, the cMADRS total score assessed at study entry, reflecting a time period of at least 4 weeks of adequate AD psychopharmacotherapy, was lower in MDD patients receiving quetiapine. Those patients augmented with quetiapine also showed higher response rates and, concurrently, lower odds for the development of TRD. It should be underscored in this regard that quetiapine administration was associated with a generally favorable disease profile, which may represent a possible explanation model for the observed trend towards better therapeutic outcome. Given the retrospective assessment of treatment response, the unequal distribution of both SGAs in our sample of MDD patients, and the fact that our results in general are of cross-sectional character and thus unsuitable for causal conclusions, a statement whether an individual SGA should be preferred over the other would be premature.

Considering comparable international evidence, it is relevant that investigations directly contrasting quetiapine and aripiprazole have not yet been executed in MDD. Results derived from a network meta-analysis showed very similar AD potency and tolerability of the examined agents, whereby quetiapine performed slightly better regarding treatment outcome but had higher discontinuation rates (Zhou et al., 2015). In terms of TRD, 4 respective studies reported positive results for aripiprazole augmentation (Strawbridge et al., 2018), whereas available evidence on the efficacy of quetiapine in this group of patients is scarce. However, quetiapine showed robust effects in MDEs occurring in the course of bipolar affective disorders that have been repeatedly associated with generally detrimental disease characteristics (Hidalgo-Mazzei et al., 2019), while therapeutic impact of aripiprazole was less convincing in this clinical condition (Bahji et al., 2020). The fact that quetiapine was previously shown to be sufficiently potent also when prescribed as monotherapy in MDD patients (Weisler et al., 2012) even in older age (Montgomery et al., 2014) might be of further importance while discussing therapeutic efficacy of both SGAs in the treatment of MDEs and especially TRD and/or difficult-to-treat depression, where potent and individually tailored AD treatments are sought.

Summarizing the methodological strengths and limitations in detail, the real-world patient population derived from in- and outpatient units of academic as well as non-academic centers in 8 European countries should be highlighted. The resultant heterogeneous clinical manifestations of MDD including the presence of additional melancholic, psychotic, atypical, and/or catatonic features, suicidality, psychiatric and somatic comorbidities, and the varying illness severity and course ranging from single to recurrent MDEs with mild, moderate, or severe extent of current depressive symptoms are very much in contrast to those of MDD patients investigated in the course of RCTs. Such clinical heterogeneity might best possibly reflect the international everyday routine and, hence, represents a major strength. Potential cross-site differences that might have arisen in the course of recruitments in different European countries and cannot be fully ruled out were, however, considered in our statistical analyses. It should be noticed in this context that the present large multi-site project conducted by the GSRD (Souery et al., 2007; Schosser et al., 2012; Bartova et al., 2019) was not originally designed to investigate individual augmentation strategies. As a consequence, the information about when the augmentation treatment was commenced and which psychopharmacotherapeutic strategy in general seemed to be pivotal is missing. Moreover, records of the exact dosages of the applied SGAs were not consistently available throughout all patients. In line with available evidence and existing approvals for SGA augmentation in MDD, exclusively peroral formulations of both SGAs were considered. Quetiapine was regarded as augmentation starting from a minimum daily dose of 100 mg to avoid enrollment of patients treated with low-dose quetiapine due to sleep disturbances, agitation, or anxiety, for instance (Dold et al., 2018). However, it is noteworthy in this context that this minimum limit of 100 mg/d is slightly less than in most RCTs largely investigating daily doses between 100 and 300 mg (Pae et al., 2010) and might, hence, represent a further limiting aspect. Moreover, we did not distinguish between the extended and immediate release formulation of quetiapine given the differences of formulation availabilities across the participating European countries. With respect to the daily dosages of aripiprazole, minimally 2.5 mg/d was mandatory for consideration as augmentation treatment, being aware of the reduced efficacy of low-dose aripiprazole (2 mg) reported previously (Fava et al., 2012) as well as the similarly diverging formulation availabilities throughout Europe. Additionally, it should be considered in this context that the information about treatment strategies employed in the course of previous MDEs, which would further elucidate the current results and their interpretation, is not available due to the implemented study design.

To warrant unrestricted comparability with already existing evidence, the psychopharmacotherapeutic terminology used in the present work is based on the traditional indication-based nomenclature that is, however, increasingly being replaced by a new classification system driven by the pharmacological profiles of the individual substances. According to the so-called neuroscience-based nomenclature that was developed to support rational and lucid prescribing with the aim to increase therapeutic adherence (Zohar et al., 2015; Frazer and Blier, 2016), the term “SGA” would be replaced by “the D2- and 5-HT2 receptor antagonist and norepinephrine re-uptake inhibitor” in case of quetiapine, while aripiprazole would be labeled “the D2- and 5-HT1a receptor partial agonist and 5-HT2 receptor antagonist.”

Most importantly, the retrospective assessment of treatment outcome performed in the course of a cross-sectional investigation should be critically considered because it is less accurate than prospective and longitudinal approaches. However, it is essential in this context that supporting international data, showing that MDD patients are able to adequately and consistently recall and report their previous depressive symptoms even 2 years thereafter (Dunlop et al., 2019), exist. Furthermore, the mandatory extensive trainings of our raters who were exclusively experienced psychiatrists aimed at reducing any biased results at a maximum possible extent.

## Conclusions

According to the present results, factors associated with a more chronic and severe profile of MDD reflected by the presence of psychiatric and somatic comorbidities and trend-wise additional psychotic features, a higher extent of depressive symptoms, the requirement of more complex psychopharmacotherapy, and worse treatment outcome during the current MDE, as well as a longer time-period spent in psychiatric inpatient-care during the lifetime, seem to encourage clinicians to choose aripiprazole over quetiapine. Theoretically, the fact that quetiapine, which was administered in the majority of our MDD patients and was generally linked to rather beneficial disease and treatment outcome patterns, currently represents the only SGA that is approved by the European Medicines Agency for augmentation in MDD might have moderated European clinicians’ readiness to enrich their psychopharmacotherapeutic armamentarium with aripiprazole, a substance beyond the current approval, preferably in MDD patients who are more severely ill. Although existing evidence similarly supports the employment of aripiprazole in TRD on the one hand (Strawbridge et al., 2018, 2019), there is a considerable number of available studies supporting the superiority of quetiapine that showed efficacy even when administered as monotherapy on the other hand (Weisler et al., 2012; Montgomery et al., 2014).

Given the mixed results in general and considering that the most current CPGs recommend augmentation with SGAs as first choice in case of insufficient treatment response in MDD (Bauer et al., 2017; Dold and Kasper, 2017) but do not advise which individual SGA might be preferred (Bayes and Parker, 2018), further research is warranted. To be able to draw valid conclusions and generate unambiguous recommendations regarding the choice of first-line SGA augmentation in MDD in future CPGs, further studies may elaborate on our findings. However, several limitations, especially the retrospective assessment of treatment response, the markedly smaller proportion of patients receiving aripiprazole augmentation generally showing an unfavorable disease profile, and the partially heterogeneous statistical robustness of our results should be considered when interpreting our findings.

## Interest Statement

Dr Bartova has received travel grants and consultant/speaker honoraria from AOP Orphan, Medizin Medien Austria, Vertretungsnetz, Schwabe Austria, Janssen, and Angelini. Dr Dold has received travel grants and consultant/speaker honoraria from Janssen-Cilag. Dr Fugger has received consultant/speaker honoraria from Janssen. Dr Zohar has received grant/research support from Lundbeck, Servier, and Pfizer; he has served as a consultant or on the advisory boards for Servier, Pfizer, Solvay, and Actelion; and he has served on speakers’ bureaus for Lundbeck, GlaxoSmithKline, Jazz, and Solvay. Dr Mendlewicz is a member of the board of the Lundbeck International Neuroscience Foundation and of the advisory board of Servier. Dr Souery has received grant/research support from GlaxoSmithKline and Lundbeck; and he has served as a consultant or on advisory boards for AstraZeneca, Bristol-Myers Squibb, Eli Lilly, Janssen, and Lundbeck. Dr Montgomery has served as a consultant or on advisory boards for AstraZeneca, Bionevia, Bristol-Myers Squibb, Forest, GlaxoSmithKline, Grunenthal, Intellect Pharma, Johnson & Johnson, Lilly, Lundbeck, Merck, Merz, *M’s Science*, Neurim, Otsuka, Pierre Fabre, Pfizer, Pharmaneuroboost, Richter, Roche, Sanofi, Sepracor, Servier, Shire, Synosis, Takeda, Theracos, Targacept, Transcept, UBC, Xytis, and Wyeth. Dr Fabbri has been supported by Fondazione Umberto Veronesi. Dr Serretti has served as a consultant or speaker for Abbott, Abbvie, Angelini, AstraZeneca, Clinical Data, Boehringer, Bristol-Myers Squibb, Eli Lilly, GlaxoSmithKline, Innovapharma, Italfarmaco, Janssen, Lundbeck, Naurex, Pfizer, Polifarma, Sanofi, and Servier. Within the last 3 years, Dr Kasper received grants/research support, consulting fees, and/or honoraria from Angelini, Celegne GmbH, Eli Lilly, Janssen-Cilag Pharma GmbH, KRKA-Pharma, Lundbeck A/S, Mundipharma, Neuraxpharm, Pfizer, Sanofi, Schwabe, Servier, Shire, Sumitomo Dainippon Pharma Co. Ltd., sun Pharma, and Takeda. All other authors declare no conflicts of interest.
